# Crystal structure of Ankyrin-G in complex with a fragment of Neurofascin reveals binding mechanisms required for integrity of the axon initial segment

**DOI:** 10.1016/j.jbc.2022.102272

**Published:** 2022-07-16

**Authors:** Liping He, Wenli Jiang, Jianchao Li, Chao Wang

**Affiliations:** 1 Department of Neurology, The First Affiliated Hospital of USTC, Ministry of Education Key Laboratory for Cellular Dynamics, Hefei National Research Center for Physical Sciences at the Microscale, Biomedical Sciences and Health Laboratory of Anhui Province, School of Life Sciences, Division of Life Sciences and Medicine, University of Science and Technology of China, Hefei, P. R. China; 2 Division of Cell, Developmental and Integrative Biology, School of Medicine, South China University of Technology, Guangzhou, P. R. China

**Keywords:** Ankyrin-G, Neurofascin, L1CAM, axon initial segment, neuronal polarity, crystal structure, L1 syndrome, ABD, ankyrin-binding domain, AIS, axon initial segment, DIV, days *in vitro*, HA, hemagglutinin, ITC, isothermal titration calorimetry, Nfasc, Neurofascin 186

## Abstract

The axon initial segment (AIS) has characteristically dense clustering of voltage-gated sodium channels (Nav), cell adhesion molecule Neurofascin 186 (Nfasc), and neuronal scaffold protein Ankyrin-G (AnkG) in neurons, which facilitates generation of an action potential and maintenance of axonal polarity. However, the mechanisms underlying AIS assembly, maintenance, and plasticity remain poorly understood. Here, we report the high-resolution crystal structure of the AnkG ankyrin repeat (ANK repeat) domain in complex with its binding site in the Nfasc cytoplasmic tail that shows, in conjunction with binding affinity assays with serial truncation variants, the molecular basis of AnkG–Nfasc binding. We confirm AnkG interacts with the FIGQY motif in Nfasc, and we identify another region required for their high affinity binding. Our structural analysis revealed that ANK repeats form 4 hydrophobic or hydrophilic layers in the AnkG inner groove that coordinate interactions with essential Nfasc residues, including F1202, E1204, and Y1212. Moreover, we show disruption of the AnkG–Nfasc complex abolishes Nfasc enrichment at the AIS in cultured mouse hippocampal neurons. Finally, our structural and biochemical analysis indicated that L1 syndrome-associated mutations in L1CAM, a member of the L1 immunoglobulin family proteins including Nfasc, L1CAM, NrCAM, and CHL1, compromise binding with ankyrins. Taken together, these results define the mechanisms underlying AnkG–Nfasc complex formation and show that AnkG-dependent clustering of Nfasc is required for AIS integrity.

Neurons are highly polarized cells typically composed of a soma, multiple dendrites, and a single axon. The specialized compartmentalization of neurons is critical for their physiological functions. The axon initial segment (AIS) connects the soma and the axon, serving as a vital region responsible for initiating an action potential and maintaining neuronal polarity (1, 2, 3). Several studies conducted over the past decades have expanded our understanding of the molecular architecture and organization of the AIS (4, 5, 6, 7). The AIS is characterized by highly dense enrichment with a variety of proteins that include ion channels, cell adhesion molecules, scaffold proteins, regulatory proteins, and cytoskeletal proteins (8, 9, 10). Among these proteins, Ankyrin-G (AnkG) is considered as the master organizer that directs the recruitment of diverse components to the AIS (4, 5). Previous studies have demonstrated that AnkG interacts with Nav1.2, a primary ion channel involved in the initiation of an action potential (11, 12), as well as with Ndel1, a dynein regulator that functions in selective sorting and polarity maintenance at the AIS (13, 14). In addition, AnkG is also well known to interact with Neurofascin 186 (Nfasc), a cell adhesion molecule that is essential for AIS integrity (15, 16). Loss of AnkG leads to disruption of axonal polarity and disassembly of the AIS, either *in vivo* or *in vitro* (3, 17).

In humans, the ankyrin family contains 3 known members, AnkR, AnkB, and AnkG, which are all ubiquitously expressed and perform nonredundant functions in most tissues (18, 19). In neurons, the AIS and nodes of Ranvier are specifically enriched with 480/270 kDa AnkG alternative splice variants (20, 21). Clustering of AIS membrane proteins, including sodium channels, potassium channels, Nfasc, and NrCAM has been proposed to depend on their specific interactions with the ankyrin repeats (ANK repeats) of AnkG (15, 22, 23). In earlier studies, by solving the crystal structures of ANK repeats in complex with its own autoinhibitory segments or Nav1.2, we proposed that ankyrins can utilize a combination of multiple binding sites in the inner groove of its ANK repeats to interact with various membrane protein targets (*e.g*., Nav1.2, Nfasc) (24, 25). However, more high-resolution structures of ankyrins in complex with their targets are needed to fully disclosure the diverse target recognition mechanisms of ANK repeats.

Nfasc is a membrane-spanning cell adhesion molecule belonging to the L1 group of the immunoglobulin superfamily (L1 family) proteins, that include L1CAM, Neurofascin, NrCAM, and CHL1 (26, 27). Nfasc is a major splicing variant that is predominantly expressed in neurons and is restricted to the AIS and nodes of Ranvier (28). Loss of Nfasc leads to dissociation of the AIS in Nfasc-null mice (29). A recent study showed that Nfasc is highly mobile after its recruitment to the axonal membrane and diffuses bidirectionally until anchored at the AIS through its interaction with AnkG (30). These findings thus highlight the importance of the Nfasc–AnkG complex in forming the AIS. Previous studies have suggested that Nfasc targeting to the AIS is dependent upon AnkG binding through a FIGQY motif in its cytoplasmic domain (31, 32). However, despite a wealth of available functional data, the mechanistic details and structural basis of their interaction remain poorly understood.

In the present study, we characterized the binding and structural interactions between AnkG and Nfasc in detail. Analysis of truncation variants showed that a segment (1187–1214) in the cytoplasmic tail of Nfasc mediates its strong interaction with AnkG. We then elucidated the precise molecular mechanisms governing this interaction by solving the crystal structure of the ankyrin-binding region of Nfasc in complex with the ANK repeats of AnkG. In addition to the recognized FIGQY motif, our structural data identified an N-terminal region that is also essential for binding. Further structural analysis highlighted a pattern of preferential distribution of residues in 4 hydrophobic or hydrophilic layers formed by ANK repeats in the inner groove responsible for targets binding. Moreover, we showed that disruption of AnkG binding abolishes Nfasc localization to the AIS in primary cultured hippocampal neurons. We also confirmed that AnkG can interact with all 4 members of the L1 family and found that 2 L1 syndrome-associated mutations in L1CAM interfere with its binding to ankyrins. These results provide inroads to understanding the possible pathological mechanisms underlying ankyrin-L1 family complex–related neuronal diseases.

## Results

### Nfasc ankyrin-binding domain in the cytoplasmic tail mediates binding with AnkG

**Figure 1:**
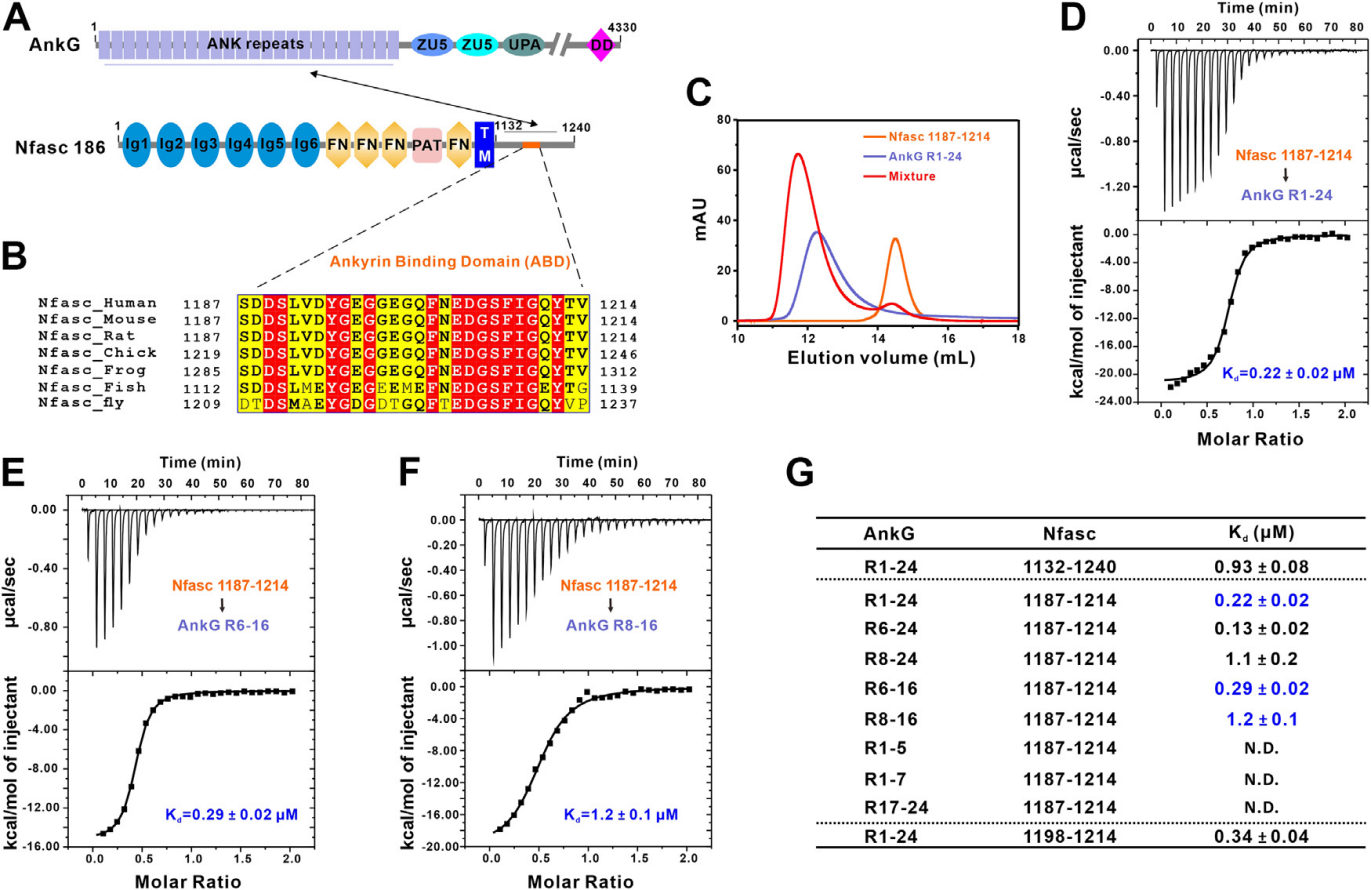
**Nfasc ABD in the cytoplasmic tail mediates binding with AnkG.***A*, schematic diagram showing the domain organizations of AnkG and Nfasc. In this drawing, the interaction between AnkG and Nfasc is indicated by a two-way arrow. The ankyrin binding domain (ABD) of Nfasc are highlighted in *orange* in the cytoplasmic region. DD, death domain. *B*, sequence alignment of Nfasc ABD in different species showing a high conservation through evolution. Residues that are identical and highly similar are indicated in *red* and *yellow boxes*, respectively. *C*, analytical gel filtration analysis showing that Nfasc ABD (residues 1187–1214) and AnkG ANK repeats (R1-24, residues 38–855) interacted with each other. *D*–*F*, ITC-based measurements of the binding affinity of Nfasc 1187 to 1214 with AnkG R1-24 (*D*), AnkG R6-16 (*E*), or AnkG R8-16 (*F*). The *K*_*d*_ error is the fitting error obtained using 1 site–binding kinetics model in Origin 7.0 to fit the ITC data. *G*, the measured binding affinities between different Nfasc fragments and various truncations of AnkG ANK repeats from ITC-based binding assays. N.D. indicates that no binding was detected. Nfasc, Neurofascin 186.

Previous studies have established that Nfasc binds to the AnkG ankyrin repeats (ANK repeats) region through interaction with its C-terminal cytoplasmic tail (15, 31). To investigate the biochemical details of this interaction, we used purified AnkG ANK repeats (residues 38–855, hereafter R1-24) and 2 fragments of Nfasc (including the full-length Nfasc cytoplasmic tail, residues 1132–1240, and the ankyrin-binding domain, ABD, residues 1187–1214) for FPLC and isothermal titration calorimetry (ITC) assays to evaluate the binding affinity and map the specific sites that participate in their interaction (Fig. 1, *A* and *B*). Both FPLC and ITC results showed that the Nfasc ABD interacts strongly with AnkG R1-24 (Fig. 1, *C* and *D*). Furthermore, Nfasc ABD is both necessary and sufficient for binding to AnkG with a dissociation constant (*K*_*d*_) of ∼ 0.22 μM (Fig. 1*G*, the first 2 rows and the last row, & Fig. S1).

Based on findings established in our previous studies of ANK repeats (24), we designed several AnkG truncation variants to identify the precise Nfasc-binding site. Through comprehensive analyses by ITC and FPLC, we found that the major binding region was located between the eighth and sixteenth ANK repeats (AnkG R8-16), while the first 7 repeats and last 8 repeats exhibited no binding activity with Nfasc (Figs. 1, *E*–*G* and S1 and S2). The binding affinity between the AnkG R6-16 and Nfasc showed a stronger trend than that of AnkG R8-16 (*K*_*d*_ ∼ 0.29 μM *versus K*_*d*_ ∼ 1.2 μM), which suggested that the 2 N-terminal repeats could possibly involve in binding (Fig. 1, *E*–*G*). Taken together, these results showed that Nfasc interacts with AnkG strongly and the ABD of the Nfasc cytoplasmic tail mediated its interactions with AnkG.

### Overall crystal structure of AnkG in complex with Nfasc ABD

**Figure 2:**
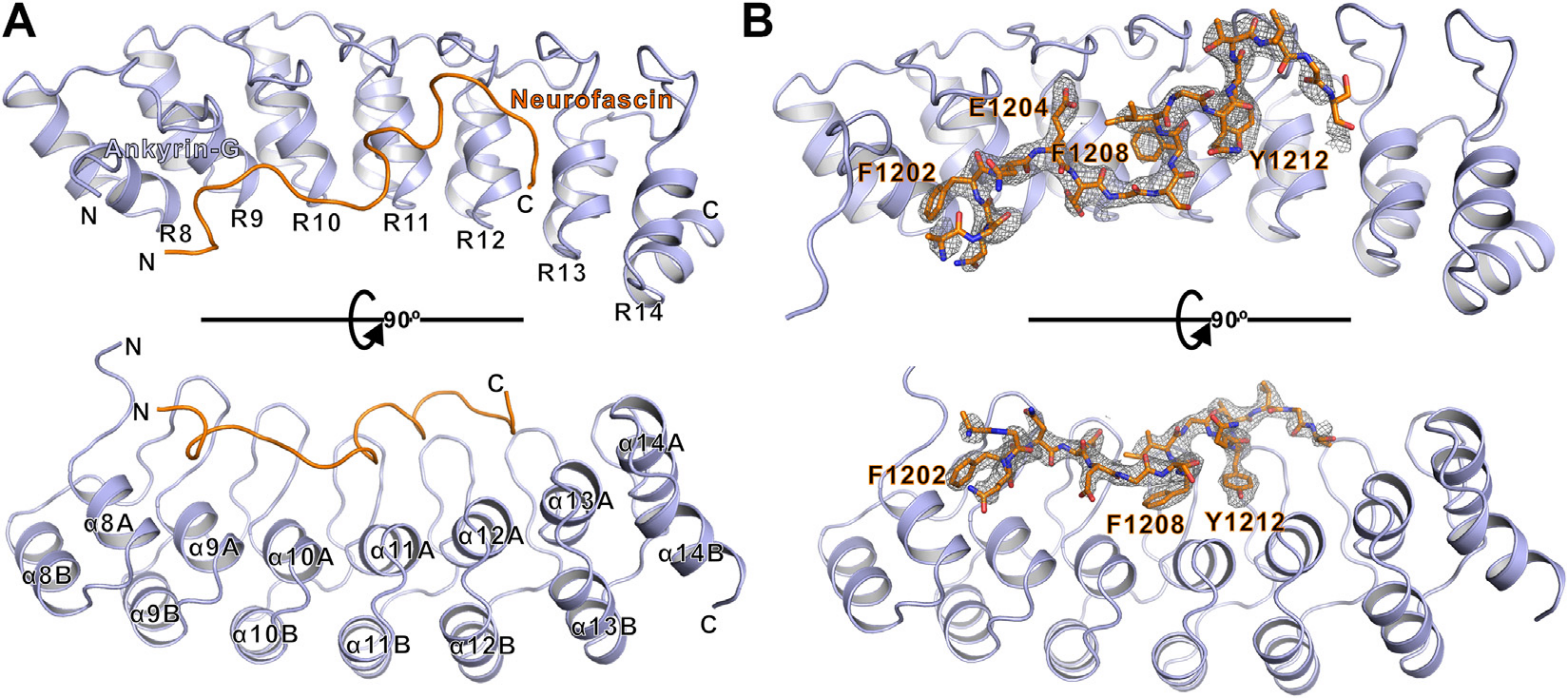
**Overall crystal structure of AnkG in complex with Nfasc ABD.***A*, ribbon representation model showing the overall structure of the AnkG–Nfasc complex. In this drawing, AnkG is shown in *light blue* and Nfasc is shown in *orange*. Nfasc peptide extends in the inner groove of ANK repeats with parallel orientation. The coordinates and structure factors of the AnkG and Nfasc complex have been deposited to the Protein Data Bank under the accession code number 7XCE. *B*, electron density of a Fo-Fc composite omit map contoured at 3 σ. The omit map was generated using the simulated annealing method by omitting the Nfasc part. AnkG is shown in ribbon and the Nfasc fitting in the electron density is displayed as sticks with the critical residues including F1202, E1204, F1208, and Y1212 highlighted. ABD, ankyrin-binding domain; Nfasc, Neurofascin 186.

In order to better understand the molecular mechanisms governing AnkG–Nfasc complex formation, we sought to solve the crystal structure of AnkG–Nfasc in complex. To this end, we tested various preparations of the protein complex or combinations of fusion proteins guided by the results of our aforementioned binding assays. After extensive efforts, we successfully obtained crystals of Nfasc ABD fused with the AnkG R8-16 region that diffracted to 2.5 Å resolution, and we subsequently solved the complex structure using molecular replacement (Fig. 2 and Table S1).

In this complex, the AnkG R8-14 adopted a canonical ANK repeat architecture (Fig. 2, *A* and *B*), although it should be noted that the last 2 ANK repeats (R15 and R16) were missing in the final structure. In agreement with our understanding of ankyrin structure, the Nfasc ABD peptide extended into the inner groove formed by the ANK repeats. However, in contrast with the antiparallel orientation (N-terminal to C-terminal binding) observed in other ANK repeats–target complex structures reported for ankyrin family proteins (Nav1.2-AnkB, AnkR-AnkB, etc.) (24), the AnkG–Nfasc complex adopted an unexpected parallel binding orientation (*i.e*., N-terminal to N-terminal binding between Nfasc and ANK repeats). Although we used the Nfasc 1187 to 1214 segment for crystallization, only the Nfasc 1198 to 1214 region could be clearly traced in the electron density map (Fig. 2*B*). Furthermore, this 1198 to 1214 region retained the majority of its binding ability with AnkG, which was in line with our findings in truncation variants (Fig. 1*G*). Given the parallel orientation combined with the aforementioned biochemical data, it was reasonable to speculate that the Nfasc 1187 to 1197 residues might facilitate binding through interaction with the N-terminal ANK repeats adjacent to R8-16 in AnkG.

### Hydrophobic and hydrogen bonding interactions at the AnkG–Nfasc interface

**Figure 3:**
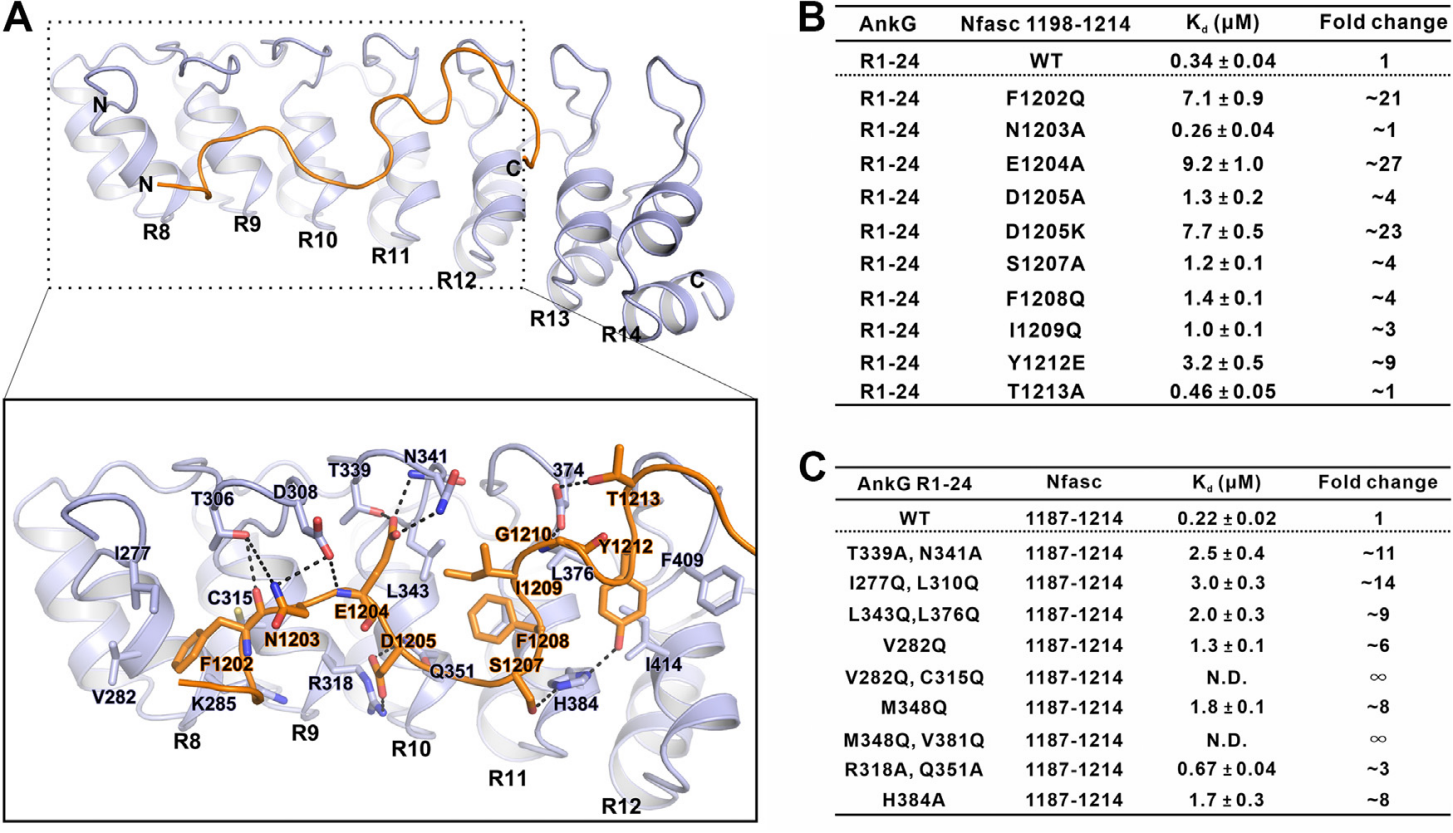
**The interface of AnkG–Nfasc complex.***A*, ribbon diagram showing the detailed interactions between AnkG and Nfasc. Residues involved in the binding are shown in stick model. The hydrogen bonds are shown as dotted lines. *B*, the measured binding affinities between various mutations of Nfasc 1198 to 1214 and AnkG R1-24 based on ITC assays and comparison against Nfasc 1198 to 1214 WT in binding with AnkG R1-24. *C*, the measured binding affinities between various mutations in each layer of AnkG R1-24 and the Nfasc 1187 to 1214 based on ITC assays and comparison against AnkG R1-24 WT in binding with Nfasc 1187 to 1214. ITC, isothermal titration calorimetry; Nfasc, Neurofascin 186.

**Figure 4:**
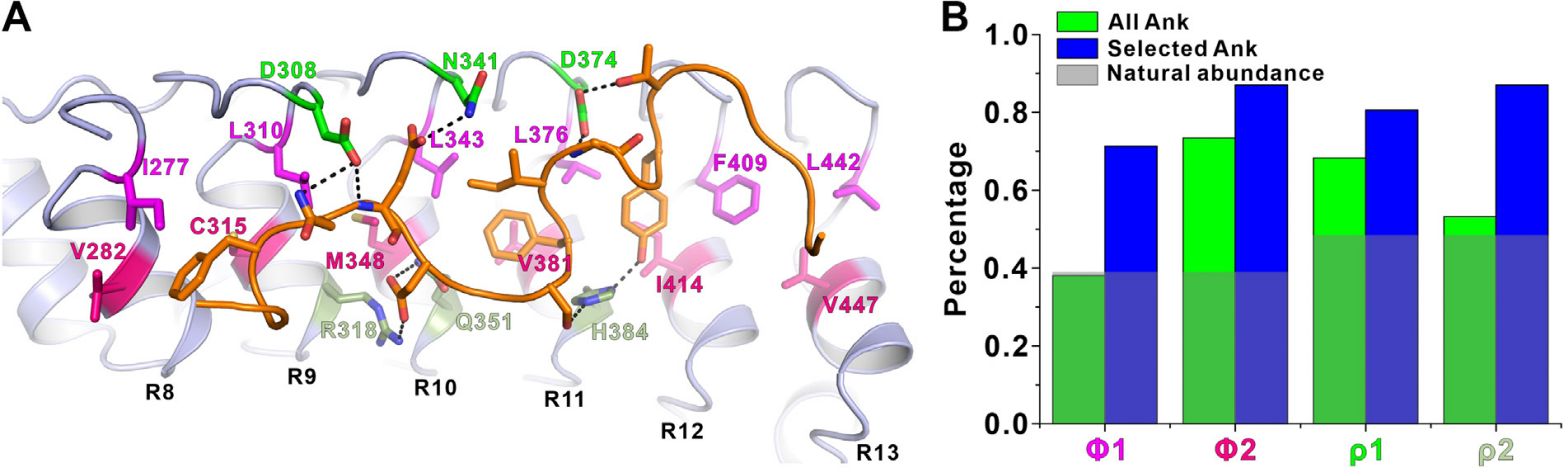
**Amino acids in ANK repeats show distribution preference in inner groove-dependent binding.***A*, the amino acids layers of ANK repeats in the interface of AnkG–Nfasc complex. Amino acids of 4 layers in ANK repeats displayed patterned distributions are highlighted in stick model. Layer 1 (*green*) and layer 4 (*pale green*) are polar residues while layer 2 (*magenta*) and layer 3 (*hot pink*) are hydrophobic residues. The hydrogen bonds are shown as dotted lines. *B*, histogram of residues distribution preference analysis of the ANK repeats in inner groove-dependent binding. *Green boxes* represent all ANK repeats. *Blue boxes* represent the ANK repeats from human AnkR/B/G, KANK1/2, Espin1, and Espin1-like proteins, which bind to their targets in the inner groove-dependent manner (some of the representative structures were shown in Fig. S5). *Gray boxes* represent the natural distribution of the residues in all human proteins. Φ1, Φ2, ρ1, and ρ2 are corresponding to layer 2, layer 3, layer 1, and layer 4 positions, respectively. The vertical axis represents the percentage of hydrophobic residues (Φ1, Φ2) or polar residues (ρ1, ρ2) in ANK repeats. Nfasc, Neurofascin 186.

We next examined the specific interactions between residues responsible for their binding and found that the AnkG–Nfasc interface is mainly mediated by hydrophobic and hydrogen bonding interactions. In particular, F1202 from Nfasc inserts into a hydrophobic pocket formed by I277, V282, and C315 from AnkG (Figs. 3*A* and 4*A*). Similarly, F1208, another aromatic residue from Nfasc, occupies the hydrophobic groove formed by AnkG residues L343, L376, and V381 (Figs. 3*A* and 4*A*). In addition to these central hydrophobic interactions, several hydrogen bonds also contribute to the high affinity and specificity of the interaction. Among these, we found that the E1204 sidechain from Nfasc forms hydrogen bonds with T339 and N341 in AnkG, while the main chain of E1204 forms another hydrogen bond with the sidechain of D308 from AnkG (Fig. 3*A*). In addition, D1205 from Nfasc forms hydrogen bonds with R318 and Q351 from AnkG (Fig. 3*A*). It should be noted that several other hydrogen bonding pairs, including T306^AnkG^-N1203^Nfasc^, H384^AnkG^-S1207/Y1212^Nfasc^, and D374^AnkG^-G1210/T1213^Nfasc^, also contribute to stabilizing the complex assembly (Fig. 3*A*). Collectively, ∼989 Å^2^ total surface area (calculated by PISA server, https://www.ebi.ac.uk/pdbe/pisa/) participated in the interaction between the AnkG ANK repeats and the Nfasc ABD peptide.

To further confirm the interaction mode between AnkG and Nfasc, we evaluated the impact of specific mutations in either AnkG or Nfasc at the binding interface. In line with our structural analysis, ITC-based assays showed that mutations in the Nfasc ABD that disrupted hydrophobic interactions generally weakened AnkG–Nfasc interaction (Fig. 3*B*). For example, an F1202Q conversion mutation in the Nfasc ABD variant partially disrupted hydrophobic interactions and led to a greater than 20-fold decrease in binding (Figs. 3*B* and S3*A*). Single substitutions of Gln at the F1208 or I1209 residues in Nfasc led to a slight decrease of their interaction (Figs. 3*B* and S3, *G* and *H*), whereas an Ala substitution at E1204 in Nfasc resulted in a significant ∼27-fold decrease in binding affinity (Figs. 3*B* and S3*C*), and D1205A/K substitutions in Nfasc variants also weakened its binding with AnkG (Fig. 3*B* and S3, *D* and *E*). These findings thus illustrated the pivotal role of these hydrogen bonds at the ABD-ANK repeat interface in the crystal structure. Notably, Nfasc residue Y1212, which is a likely phosphorylation site, was reported to act as a molecular switch for regulating the interaction between Nfasc and AnkG (32). However, we found that a Y1212E phosphorylation mimic variant exhibited only an approximately 9-fold decrease in binding, potentially due to incomplete imitation of the *in vivo* effects of phosphorylation (Figs. 3*B* and S3*I*). Overall, the observed hydrophobic and hydrogen bonding/electrostatic interactions between AnkG and Nfasc strongly resembled those interactions between the inner groove of ankyrins and short peptides of other targets described in previous studies (24, 25).

### Amino acids in ANK repeats show distribution preference in inner groove-dependent binding

Next, we evaluated the specific roles of AnkG residues required for binding with Nfasc in the inner groove. With great interest, we found that residues in the ANK repeats located in inner groove exhibited clear patterns of distribution to form distinct hydrophilic layers and hydrophobic layers (Fig. 4*A*). More specifically, polar residues from the upper loop connecting 2 adjacent ANK repeats (D308, N341, and D374) formed the first, hydrophilic, layer (*green*). The second, hydrophobic, layer (*magenta*) consisted of residues from the hairpin region, including I277, L310, L343 L376, F409, and L442. Residues from the belly of the αA (V282, C315, M348, V381, I414, and V447) formed the third, hydrophobic, layer (*hot pink*), while residues from the bottom of αA (R318, Q351, and H384) formed the fourth, hydrophilic, layer (*pale green*) (Fig. 4*A*). Obviously, residues in the hydrophilic layers participated in the electrostatic or hydrogen bond interactions with N1203, E1204, D1205, S1207, Y1212, and T1213 from Nfasc, and residues in the hydrophobic layers were responsible for coordination of hydrophobic residues of Nfasc, including F1202, F1208, and I1209 (Figs. 3*A* and 4*A*).

We then introduced corresponding mutations into each hydrophilic or hydrophobic layer of AnkG and, consistent with these predicted functions, ITC assays showed that all of these mutations could decrease the binding affinity for the Nfasc ABD (Figs. 3*C* and S4). Among them, double substitutions for Gln in the hydrophobic residues of the hydrophobic third layer completely abolished binding with the Nfasc ABD, indicating that these residues in the third layer were indispensable for AnkG–Nfasc interactions (Figs. 3*C* and S4, *E* and *G*).

We found that in several ANK repeats containing proteins (AnkR/B/G, KANK1/2, Espin1/Espin like) that bind their respective target peptides in an inner groove-dependent manner, they all utilize these 4 layers of residues for binding. We then wondered whether there were amino acid preferences in these 4 layers of ANK repeats for targets binding. In Φ1 and Φ2 (corresponding to layer 2 and 3) positions, we observed an obvious preference for hydrophobic amino acids (A/C/F/I/L/M/V/W/Y) (Figs. 4*B* and S5). Particularly, ∼70% of the Φ1 position of these target binding ANK repeats contains a hydrophobic residue (*blue bars*), whereas the percentages for this position in all ANK repeats in human proteome (*green bars*) or the natural abundancy of hydrophobic residues (*gray bars*) are both ∼40%. Similarly, in the ρ1 and ρ2 (corresponding to layers 1 and 4) positions, polar amino acids (D/E/H/K/N/Q/R/S/T) occurred at higher frequencies than they did in all ANK repeats or throughout the whole proteins (Figs. 4*B* and S5). Collectively, these patterns of preferential residue distribution within the 4 layers indicated that ANK repeats may share similar mechanisms for binding to diverse peptides mediated by the inner groove. Thus, we propose that by analyzing the amino acid properties for these 4 positions of ANK repeats from a protein, we might be able to determine whether this protein can use the inner groove for target recognition.

### Disruption of AnkG binding abolishes Nfasc enrichment at the AIS

**Figure 5:**
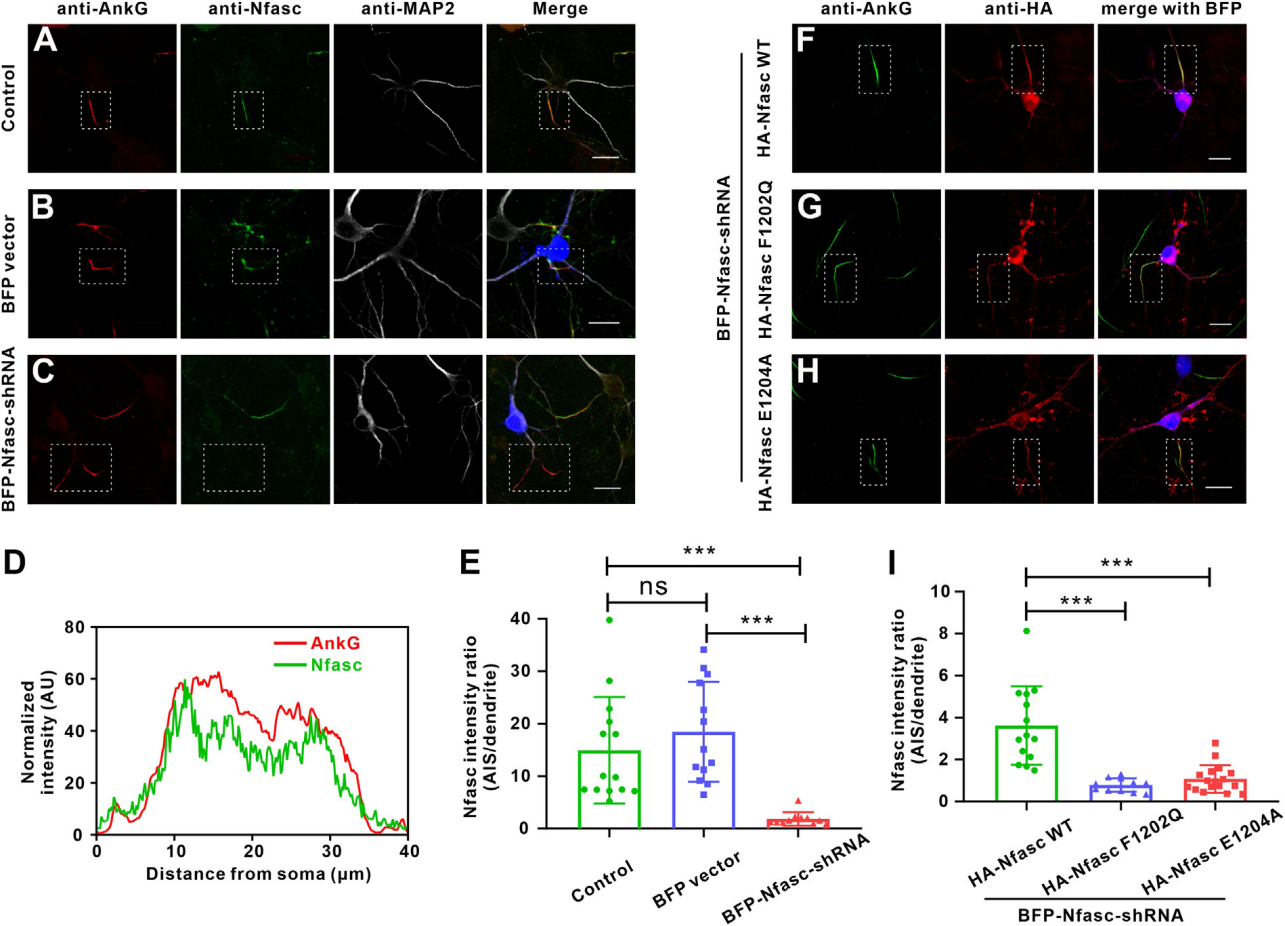
**Disruption of AnkG binding abolishes Nfasc enrichment at the AIS.***A*–*C*, WT neurons (*A*) or neurons transfected with BFP vector (*B*), or BFP-Nfasc-shRNA (*C*) and stained for AnkG (*red*), Nfasc (*green*), and MAP2 (*white*). Nfasc-shRNA significantly decreased Nfasc (*green*) signals in cultured hippocampal neurons. AnkG (*red*) marks the AIS and MAP2 (*white*) marks dendrites. The AIS region is marked by dotted boxes. The scale bar represents 20 μm. *D*, fluorescence intensity plots of panel (*A*) provide a comparison of the immunosignal strength of AnkG (AIS, *red*) and Nfasc (*green*), showing the colocalization at the AIS. *E*, quantification of Nfasc fluorescence intensity in Nfasc-shRNA transfected neurons (n = 11) compared with the BFP vector transfected neurons (n = 13) and WT neurons (n = 14). N value represents the number of neurons selected in 3 batches of neuronal preparations. ∗∗∗*p*＜0.001; ns, not significant. The student’s *t* test is performed. Error bars, SEM. *F*, the shRNA-resistant HA-Nfasc-WT effectively restored the enrichment at the AIS. BFP marks the Nfasc-shRNA transfected neurons. The AIS region is marked by dotted boxes. The scale bar represents 20 μm. *G* and *H*, the HA-Nfasc-F1202Q (*G*) or HA-Nfasc-E1204A (*H*) failed to enrich at the AIS. BFP marks the Nfasc-shRNA transfected neurons. The AIS region is marked by dotted boxes. The scale bar represents 20 μm. *I*, quantification of Nfasc fluorescence intensity in neurons recused with HA-Nfasc-WT (n = 14), HA-Nfasc-F1202Q (n = 11), or HA-Nfasc-E1204A (n = 17). N value represents the number of neurons selected in 3 batches of neuronal preparations. ∗∗∗*p*＜0.001; ns, not significant. The student’s *t* test is performed. Error bars, SEM. AIS, axon initial segment; HA, hemagglutinin; Nfasc, Neurofascin 186.

Both AnkG and Nfasc are known to specifically localize at the AIS region in neurons in mice and humans (29, 33, 34). Consistent with these previously reported data, we first confirmed that AnkG and Nfasc both showed clear enrichment and colocalization at the AIS in primary cultured hippocampal neurons obtained from C57BL/6 mice (Fig. 5, *A* and *D*). To investigate the function of AnkG–Nfasc complex in neurons, we suppressed endogenous Nfasc expression using Nfasc-shRNA at day 4 of *in vitro* in hippocampal neuron culture (days *in vitro* [DIV] 4). At DIV 7, immunofluorescent staining showed that Nfasc signal was substantially reduced in neurons transfected with BFP-Nfasc-shRNA compared to those transfected with the BFP vector (Fig. 5, *B*, *C*, *E*). Interestingly, the intensity of AnkG signal did not change between DIV 4 and DIV 7, which indicated that enrichment for AnkG at the AIS did not depend on Nfasc at this stage. We then overexpressed an hemagglutinin (HA)-Nfasc (*i.e.*, WT) complementation plasmid or an HA-Nfasc variant plasmid harboring either F1202Q or E1204A conversions in the Nfasc-depleted hippocampal neurons. We found that the WT Nfasc expression restored AIS enrichment while the F1210Q and E1204A Nfasc variants exhibited total loss of enrichment at the AIS (Fig. 5, *F*–*I*), which was in agreement with our aforementioned AnkG-binding assays. Taken together, these results indicated that the Nfasc localization to the AIS depends on its binding with AnkG and that disruption of this interaction results in Nfasc failure to localize at the AIS in neurons.

### L1 syndrome-associated mutations of L1CAM impair AnkG binding

**Figure 6:**
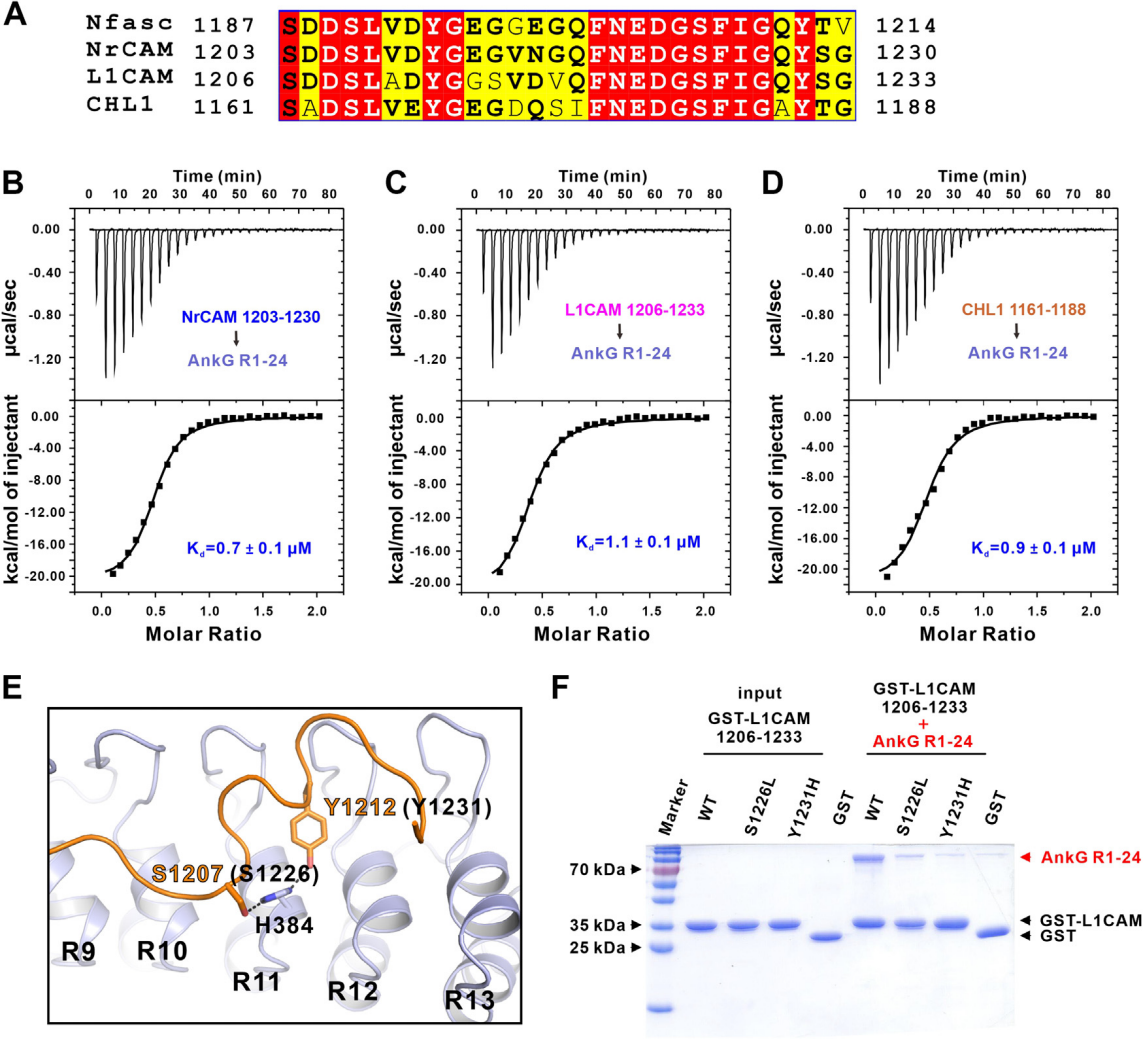
**L1 syndrome-associated mutations of L1CAM impair AnkG binding.***A*, sequence alignment of the ABD region in L1 family members including Nfasc, NrCAM, L1CAM, and CHL1. Residues that are identical and highly similar are indicated in *red* and *yellow boxes*, respectively. *B*–*D*, ITC-based measurements of the binding affinity of AnkG R1-24 with NrCAM (*B*), L1CAM (*C*), or CHL1 (*D*), showing all the L1 family proteins can bind to AnkG. *E*, structure model of Nfasc and AnkG showing the residues of Nfasc (S1207 and Y1212) corresponding to the L1 syndrome-associated residues of L1CAM (S1226 and Y1231). The residue H384 from AnkG is also highlighted in stick model. The hydrogen bonds are shown as dotted lines. *F*, GST pull-down assays showing that the S1226L and Y1231H variants of L1CAM significantly decrease the binding with AnkG R1-24. Nfasc, Neurofascin 186.

Nfasc belongs to the L1 superfamily of cell adhesion molecules that includes 3 other family members, L1CAM, NrCAM, and CHL1 (35). Earlier studies have suggested that all 4 L1 family members could form complexes with ankyrin family scaffold proteins, most likely through interactions with their respective cytoplasmic tails (15). We confirmed that the proposed Nfasc ABD region in the cytoplasmic tail was highly conserved among L1 members through sequence alignments (Fig 6*A*) and further demonstrated through ITC assays that all L1 proteins could bind to AnkG with high affinity (Fig. 6, *B*–*D*). Interestingly, through investigation of the literatures, we found that 2 mutations (S1226L and Y1231H conversions) in the L1CAM ABD (illustrated in Fig. 6*E*) have been linked to L1 syndrome, an inherited mild to severe congenital disorder characterized by corpus callosum hypoplasia, retardation, adducted thumbs, spastic paraplegia, and hydrocephalus (36, 37, 38).

In our solved crystal structure, the corresponding Ser and Tyr residues of Nfasc form 2 hydrogen bonds with H384 from AnkG (Fig. 6*E*). To evaluate whether the 2 L1 syndrome-related mutations of L1CAM affect its binding with AnkG, we performed both pull-down and ITC assays to examine their interactions. The results indicated that these 2 disease-associated mutations indeed resulted in decreased or disrupted binding to AnkG, which could explain the potential pathological mechanism of ankyrin-related trafficking or stabilization of these membrane proteins through formation of membrane microdomain structures (Figs. 6*F*, and S6). Taken together, these data suggested that the mode of AnkG–Nfasc interaction revealed in the aforementioned data could provide a structural basis for understanding ankyrin-L1 family binding.

## Discussion

The specific localization and molecular composition of the AIS enable its function of initiating an action potential and maintaining axonal polarity. AnkG has been proposed to serve as a central coordinator of AIS organization through its capacity to link diverse membrane proteins with cytoskeletal proteins (3, 4, 10, 39). In this study, we systematically characterized the detailed interactions between AnkG and Nfasc by solving the AnkG–Nfasc complex structure and identifying the residues that are essential for binding between Nfasc and AnkG. Moreover, we confirmed Nfasc is enriched at the AIS in a manner dependent on AnkG binding, thus demonstrating a role for Nfasc–AnkG complex formation in maintaining AIS integrity. Finally, we found that mutations in L1CAM linked to L1 syndrome decrease or disrupt its interactions with AnkG, suggesting that interference with ankyrin-related complex function can contribute to the pathogenesis of neuronal diseases.

Our earlier studies have shown that the N-terminal 24 ANK repeats of ankyrins form an elongated, left-handed helical solenoid structure with the αAs and hairpin loops forming a concave inner groove to coordinate diverse membrane targets binding (24). Furthermore, we demonstrate that AnkG binds to Nfasc through this inner groove of ANK repeats in the present study. Although more than a dozen of membrane binders have been reported to bind with ankyrin family members, the structural information are still very limited. Here, in the solved complex structure, AnkG reserves the typical ANK repeat architecture while Nfasc peptide extends in the elongated inner groove of the ANK repeats, showing the critical role of the inner groove. Interestingly, Nfasc is the first targets proved to interact with ANK repeats in a parallel binding orientation among the reported inner groove–dependent bindings. The crystallization was facilitated using a linker with 8 residues (“GSLVPRGS”) to covalently link the Nfasc ABD to the N-terminal of AnkG R8-14. The linker was supposed to be long enough if forming an intramolecular antiparallel interaction, as estimated in other ankyrins–targets complex structures. Unexpectedly, we found that instead of the more easily formed antiparallel intramolecular interaction, the fused Nfasc ABD bound to another AnkG molecule from the neighboring asymmetric unit, forming an intermolecular interaction (Fig. S7). Thus, we believed that the parallel binding orientation was not induced by the short covalent linkage but represented the native binding mode.

We further analyzed the amino acids in the inner groove of ANK repeats. Of note, these residues present patterns of preferential distribution to form distinct 4 layers when the inner groove is critical for targets recognition (Fig. 4). Coordination of these hydrophobic or polar residues layers endows ANK repeats with abilities to bind diverse targets, thus making the ANK repeats an ideal protein–protein interaction module. Furthermore, AnkG interacts with Nav channels mainly through the ANK repeats R1-5 and with Nfasc mainly through ANK repeats R8-12, respectively. Diversity of the binding sites usage for the membrane targets (*e.g.*, Nav channels and Nfasc) and the autoinhibitory segments from ankyrins (*e.g.*, AnkR_AS interacts with ANK repeats R1–14, AnkB_AI-b interacts with ANK repeats R1–5, and AnkB_AI-c interact with ANK repeats R17–24 (24, 25)) may indicate potential mechanisms for the regulation of the site selectivity.

Nfasc is essential for the integration of the AIS and thought to be located to the AIS with AnkG at an early stage in the axon differentiation and development period (4, 33, 39, 40). Previous studies have reported that Nfasc is highly mobile when transported to the axonal membrane (30). During moving to the proximal axon *via* retrograde transport driven by TRIM46-labeled microtubules, Nfasc is retained at the AIS by interacting with AnkG (30, 41). The AnkG–Nfasc structure solved in this study provides the structural basis of this crucial complex at the AIS and reveals a new region of Nfasc, which is critical for AnkG interaction besides the FIGQY motif (Fig. 3*A*). Our biochemical data identified the essential residues (F1202 and E1204) that significantly impact on AnkG binding (Fig. 3*B*). Moreover, data from cultured hippocampal neurons also confirmed that interference with the AnkG binding in this region of Nfasc failed to rescue Nfasc enrichment at the AIS in neurons (Fig. 5, *F*–*I*), showing that the AIS localization of Nfasc depends on its binding with AnkG. Interestingly, Nfasc depletion had no obvious influence on AnkG localization at the AIS in the earlier stage (DIV 4 to DIV 7). However, there are studies reported that shRNA-mediated knockdown of AnkG membrane partners Nav or Nfasc led to a decrease of AnkG concentration and perturbed the AIS formation and maintenance at DIV 14 (33, 42), suggesting that Nfasc indeed plays a vital role in the maintenance of the AIS architecture in a relatively longer period of time in neuronal polarity maintenance. Moreover, NF155, a glial type of Nfasc, is required for myelinating glial cells to organize the paranodal domain. Genetic ablation of NF155 results in the disruption of the paranodal axoglial junctions (43, 44). AnkB, expressed by Schwann cells, and AnkG, expressed by oligodendrocytes, are highly enriched at the glial side of the paranodal junctions where they interact with NF155 (45). Many studies have shown that glial ankyrins facilitate node formation and conditional KO of ankyrins in oligodendrocytes disrupts paranodal junction assembly (45, 46), indicating the essential role of Nfasc–ankyrin complex in the nervous system.

Previous studies have established that the L1 family proteins function together with ankyrins in diverse membrane microdomains (19). We wonder whether our findings on Nfasc–AnkG interaction could be applicable to other L1 members. Our sequence alignment and ITC data clearly showed that all L1 family proteins could bind to AnkG (Fig. 6, *A*–*D*). More importantly, we found that 2 L1 syndrome associated mutations in L1CAM ABD compromise the binding with AnkG through our structural model and glutathione-*S*-transferase (GST) pull-down experiments (Fig. 6, *E* and *F*). In addition, many studies have shown that L1CAM provides linkage to the actin cytoskeleton through AnkB in axons (47, 48, 49). Given the high similarity between AnkG and AnkB, we believe that the mutations will also impair L1CAM’s binding to AnkB and our structure may shed light on other functional complex formation beyond the specific AIS region.

In summary, our study establishes the molecular and structural basis for understanding complex formation of AnkG–Nfasc at the AIS, with implications for the maintenance of the AIS integrity and insights into the mechanisms for neuronal diseases.

## Experimental procedures

### Constructs, protein expression, and purification

All of the protein constructs were cloned into a modified pET32a vector for protein expression and confirmed by DNA sequencing. The fusion constructs used for crystal screening were made by two-step PCR with a linker sequence of “GSLVPRGS” between Nfasc ABD and AnkG ANK repeats. In particular, the construct for solving the crystal structure is made through fusing the Nfasc 1187 to 1214 (Nfasc ABD) at the N-terminal of AnkG 275 to 571 (AnkG R8-16) with the aforementioned linker sequence. The variant constructs were made by site-directed mutagenesis or standard PCR-based methods and confirmed by DNA sequencing. All the proteins were expressed in BL21 (DE3) *Escherichia coli* cells. The N-terminal thioredoxin-His_6_–tagged proteins were purified using Ni-NTA agarose affinity column followed by size-exclusion chromatography (Superdex 200 column, GE Healthcare) in the buffer containing 50 mM Tris–HCl, 1 mM EDTA, 1 mM DTT, and 100 mM NaCl or 250 mM NaCl as required at pH 7.8. For crystal screening proteins, the thioredoxin-His_6_ tag was removed by incubation with HRV 3C protease at 4 °C overnight and separated by size-exclusion chromatography.

### ITC assay

ITC measurements were carried out on a VP-ITC MicroCal calorimeter (Malvern) at 25 °C. All proteins were dissolved in the buffer containing 50 mM Tris, 100 mM NaCl, 1 mM EDTA, and 1 mM DTT at pH 7.8. Nfasc proteins (200 μM) were loaded into the syringe, and AnkG proteins (20 μM) were loaded in the cell. Each titration point was obtained by injecting a 10 μl aliquot of syringe protein into the cell at a time interval of 180 s to ensure that the titration peak returned to the baseline. The titration data were analyzed using the program Origin 7.0 (Microcal) and fitted by the one-site binding model to determine the binding affinities of Nfasc fragments with ANK repeats.

### FPLC assay

Analytical gel filtration chromatography was carried out on an AKTA Pure system (GE Healthcare). Proteins were loaded onto a Superose 12 column (GE Healthcare) or a Superdex 200 increase column equilibrated with a buffer containing 50 mM Tris, 100 mM NaCl, 1 mM EDTA, and 1 mM DTT at pH 7.8. All the graphs here were drawn by GraphPad Prism 8 (GraphPad Software).

### GST pull-down assay

For GST pull-down assays, GST-tagged L1CAM 1206 to 1233 WT, S1226L, and Y1231H variant proteins were purified in the buffer containing 50 mM Tris, 100 mM NaCl, 1 mM EDTA, and 1 mM DTT at pH 7.8 and detected by SDS-PAGE and Coomassie blue staining. The purified AnkG R1-24 proteins (60 μM) were incubated with 20 μM of various GST, GST-L1CAM 1206 to 1233 WT, GST-L1CAM 1206 to 1233 S1226L variant, or GST-L1CAM 1206 to 1233 Y1231H variant for 1 h at 4 °C. The 30 μl GSH-sepharose 4B slurry beads in the protein purified buffer were than incubated with the protein mixture for 30 min at 4 °C. After 3 times wash with the protein purify buffer, the captured proteins were eluted by 20 μl SDS-PAGE loading dye and detected by Coomassie blue staining.

### Protein crystallization and structure determination

Crystals of the AnkG–Nfasc complex were obtained by the hanging drop vapor diffusion method at 16 °C under the condition of 25% (w/v) phosphoenolpyruvate 5/4 PO/OH, 100 mM Hepes (pH 7.5). About 25% glycerol was added as the cryoprotectant before diffraction data collection. The diffraction data were collected at Shanghai Synchrotron Radiation Facility at 100 K and processed using HKL3000.

The structure was solved by PHASER software (https://www.ccp4.ac.uk/html/phaser.html) (50) using molecular replacement method with the structure of AnkB R8-14 (Protein Data Bank: 5Y4E) as the search model. The model of Nfasc was manually built according to the difference electron density map in COOT (https://www2.mrc-lmb.cam.ac.uk/personal/pemsley/coot/) (51). Further model modifications and refinements were repeated alternatively using COOT software and PHENIX software (http://www.phenix-online.org/). The final model was validated using MolProbity (52) and the statistics are shown in Table S1. The structure figures were made using PyMol software (https://pymol.org/2/).

### Hippocampal neuronal culture and transfection

Animal work was conducted in accordance with the guidelines of the University of Science and Technology of China and the Animal Care and Use Committee. The animal ethics review approval number is USTCACUC1801002. Primary hippocampal neurons were obtained from newborn C57BL/6 mice hippocampus. For each batch of neuronal culture preparation, ∼10 fresh hippocampal tissues from a litter of 5 to 7 mouse pups were digested with 0.25% trypsin (Life Technologies), and the digestion was terminated by adding 10% fetal bovine serum (HyClone). The mixture was titrated using a pipette, filtered through a 70 μm sterilized filter, and then centrifuged. The pellet was resuspend gently using Dulbecco's modified Eagle's medium (Life Technologies) added with 1% fetal bovine serum. Cells were then plated on poly-L-lysine (Sigma–Aldrich) coated glass coverslips in culture dish at a density of 5 × 10^4^ cells/ml. Neurons were incubated at 37 °C in neurobasal medium (Life Technologies) supplemented with B27, 0.5 mM glutamine, 12.5 μM glutamate and 1 × Pen Strep, and with 5% circulating CO_2_. The medium was changed every 48 h. The shRNA of Nfasc was cloned into a BFP-pll3.7 vector with the sequence 5′-CATCATTCCAACCGTCGTACT-3′ targeting mice Nfasc. The rescue plasmids of Nfasc were cloned into a HA-tagged vector. Hippocampal neurons were transfected by shRNA or rescue plasmids on day 4 using the calcium phosphate–DNA coprecipitation method. On day 7, the neurons were fixed and processed for immunostaining.

### Antibodies and immunofluorescence imaging

Mouse monoclonal antibodies against AnkG (1:1000, N106/36 NeuroMab), rabbit polyclonal antibodies against Neurofascin (1:500, Abcam ab31457), rabbit polyclonal antibodies against HA tag (1:1000, ProteinTech), and chicken polyclonal antibodies against MAP2 (1:10,000, Abcam) were used in the immunofluorescence imaging. Secondary goat antibodies conjugated to Alexa Fluor 488, 568, or 647 (ThermoFisher) were used at 1:1000 dilutions.

The cultured hippocampal neurons were fixed for 10 min at room temperature (RT) with 4% paraformaldehyde (BBI), permeabilized using 0.2 Triton X-100 (BBI) in PBS for 10 min, and blocked with blocking buffer (1% bovine serum albumin, 0.1% Tween 20; BBI). Primary antibodies were diluted in blocking buffer and incubated overnight at 4 °C. The second antibodies were also diluted in blocking buffer and incubated for 1 to 2 h at RT. All cells were washed with PBST (0.1% Tween 20 in PBS) for 3 min every time.

All the images in this study were captured using a Zeiss LSM 710 laser-scanning confocal microscope (Zeiss). The hippocampal neurons were captured using a 63 × 1.4 oil objective. The AIS is defined by the AnkG staining using anti-AnkG antibody. Soma and dendrites were indicated by MAP2 staining using anti-MAP2 antibody. Fluorescence intensity analysis was processed using ImageJ software (https://imagej.net/software/imagej/). The intensity ratios in neurons were quantified and analyzed using GraphPad Prism 8.

## Data availability

All data needed to evaluate the conclusions in the article are present in the article and/or the Supplementary Materials. The atomic coordinates of the AnkG–Nfasc complex have been deposited to the Protein Data Bank under the accession code: 7XCE.

## Supporting information

This article contains Supporting information (52, 53).

## Conflict of interest

The authors declare that they have no conflicts of interest with the contents of this article.
