# Supplementary material for: Crystal structure of Ankyrin-G in complex with a fragment of Neurofascin reveals binding mechanisms required for integrity of the axon initial segment
Source: J Biol Chem. 2022 Jul 16;298(9):102272. doi: 10.1016/j.jbc.2022.102272 (PMC9396398; doi:10.1016/j.jbc.2022.102272)
Supplement: Supplementary Table S1 and Figures S1–S7 [file mmc1.docx]

**Supplementary Materials for**

**Crystal structure of Ankyrin-G in complex with a fragment of Neurofascin reveals binding mechanisms required for integrity of the axon initial segment**

Liping He^1^, Wenli Jiang^1^, Jianchao Li^2*^, and Chao Wang^1*^

^1^Department of Neurology, the First Affiliated Hospital of USTC, Ministry of Education Key Laboratory for Cellular Dynamics, Hefei National Research Center for Physical Sciences at the Microscale, Biomedical Sciences and Health Laboratory of Anhui Province, School of Life Sciences, Division of Life Sciences and Medicine, University of Science and Technology of China, Hefei 230027, P. R. China.

^2^Division of Cell, Developmental and Integrative Biology, School of Medicine, South China University of Technology, Guangzhou 510006, P. R. China.

*Running title:* Structure of Ankyrin-G in complex with Neurofascin 186

*Corresponding authors.

Email: Chao Wang (cwangust@ustc.edu.cn); Jianchao Li (lijch@scut.edu.cn)

# Tables

**Table S1 Statistics of X-ray Crystallographic Data Collection and Model refinement**

| \| **Data collection** \|  \| \| --- \| --- \| \| Data sets \| AnkG/Nfasc \| \| Space group \| *P4_1_2_1_1* \| \| Wavelength (Å) \| 0.97915 \| \| Unit Cell Parameters (Å) \| a=b=97.877, c=89.101  α=β=γ=90° \| \| Resolution range (Å) \| 50-2.50 (2.54-2.50) \| \| No. of unique reflections \| 15440 (760) \| \| Redundancy \| 5.4 (5.6) \| \| I/σ \| 21.7 (2.7) \| \| Completeness (%) \| 99.6 (99.9) \| \| R_merge_ ^a^ (%) \| 12.4 (91.3) \| \| CC_1/2_ (last resolution shell) ^b^ \| 0.786 \| |
| --- | --- | --- | --- | --- | --- | --- | --- | --- | --- | --- | --- | --- | --- | --- | --- | --- | --- | --- | --- | --- | --- | --- | --- | --- |
| **Structure refinement** |
| \| Resolution (Å) \| 50-2.50 (2.59-2.50) \| \| --- \| --- \| \| R_cryst_ ^c^/R_free_ ^d^ (%) \| 19.10/23.70 (25.94/31.69) \| \| rmsd bonds (Å) / angles (°) \| 0.008 / 1.018 \| \| Average B factor (Å^2^) ^e^ \| 51.6 (overall)  49.9 (AnkG), 48.5 (Nfasc), 98.3 (linker) \| \| Molprobity score \| 1.32 \| \| No. of atoms \|  \| \| Protein atoms \| 1800 \| \| Water \| 30 \| \| Ligands \| 11 (SO_4_^2-^ and glycerol) \| \| No. of reflections \|  \| \| Working set \| 14671 (1427) \| \| Test set \| 744 (72) \| \| Ramachandran plot regions ^d^ \|  \| \| Favored (%) \| 98.77 \| \| Allowed (%) \| 1.23 \| \| Outliers (%) \| 0 \| |

Numbers in parentheses represent the value for the highest resolution shell.

a. R_merge_ = Σ |*I_i_* - <*I*>| / Σ*I_i_*, where *I_i_* is the intensity of measured reflection and <*I*> is the mean intensity of all symmetry-related reflections.

b. CC_1/2_ were defined by Karplus and Diederichs (53).

c. R_cryst_=Σ||*F*_calc_| – |*F*_obs_||/Σ*F*_obs_, where *F*_obs_ and *F*_calc_ are observed and calculated structure factors.

d. R_free_= Σ_T_||*F*_calc_| – |*F*_obs_||/Σ*F*_obs_, where T is a test data set of about 5% of the total unique reflections randomly chosen and set aside prior to refinement.

e. B factors and Ramachandran plot statistics are calculated using MOLPROBITY (52).

# Figures

**
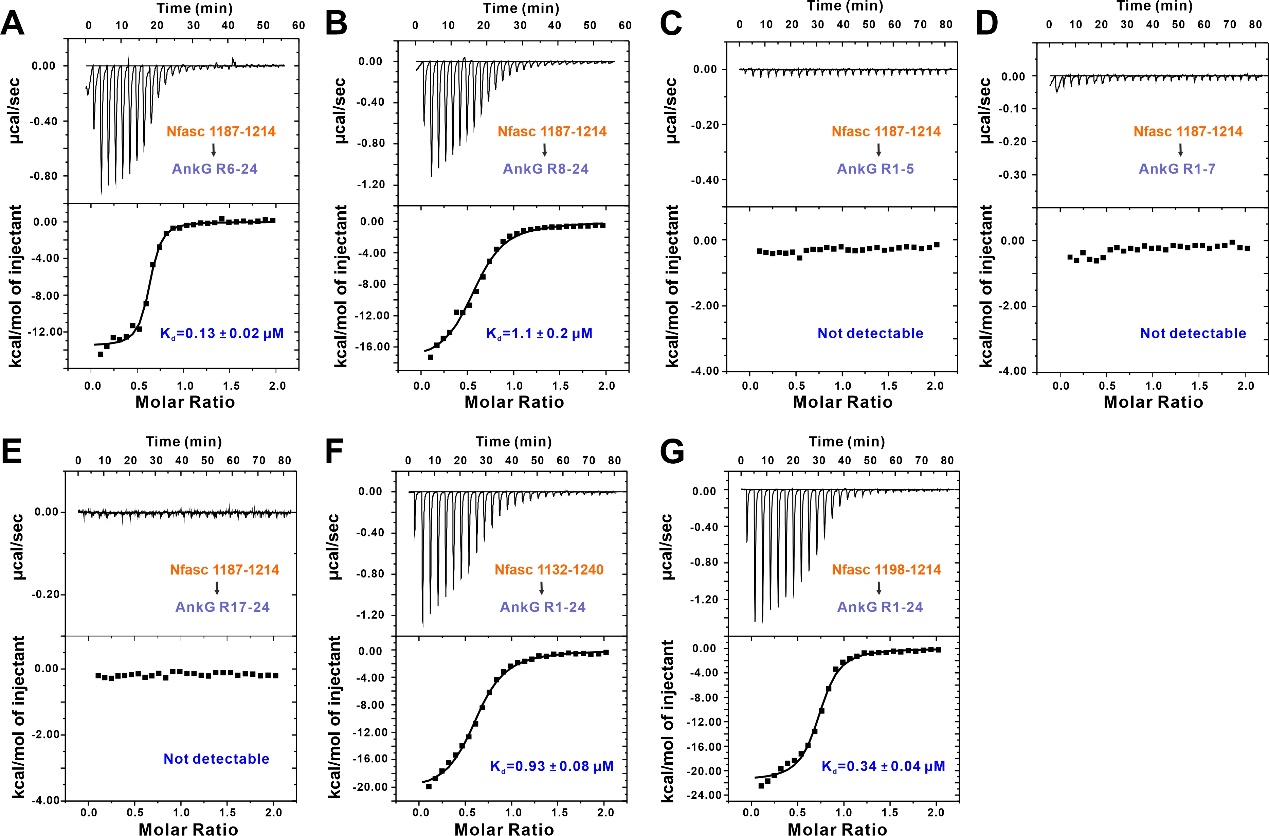
**

**Fig. S1** **The binding region mapping between AnkG and Nfasc.** (A-E) ITC-based measurements of the binding affinities of Nfasc 1187-1214 and various AnkG ANK repeats including R6-24 (A), R8-24 (B), R1-5 (C), R1-7 (D), and R17-24 (E). (F, G) ITC-based measurements of the binding affinities between different Nfasc fragments and AnkG R1-24. The K_d_ error is the fitting error obtained using one site binding kinetics model in Origin 7.0 to fit the ITC data.

**
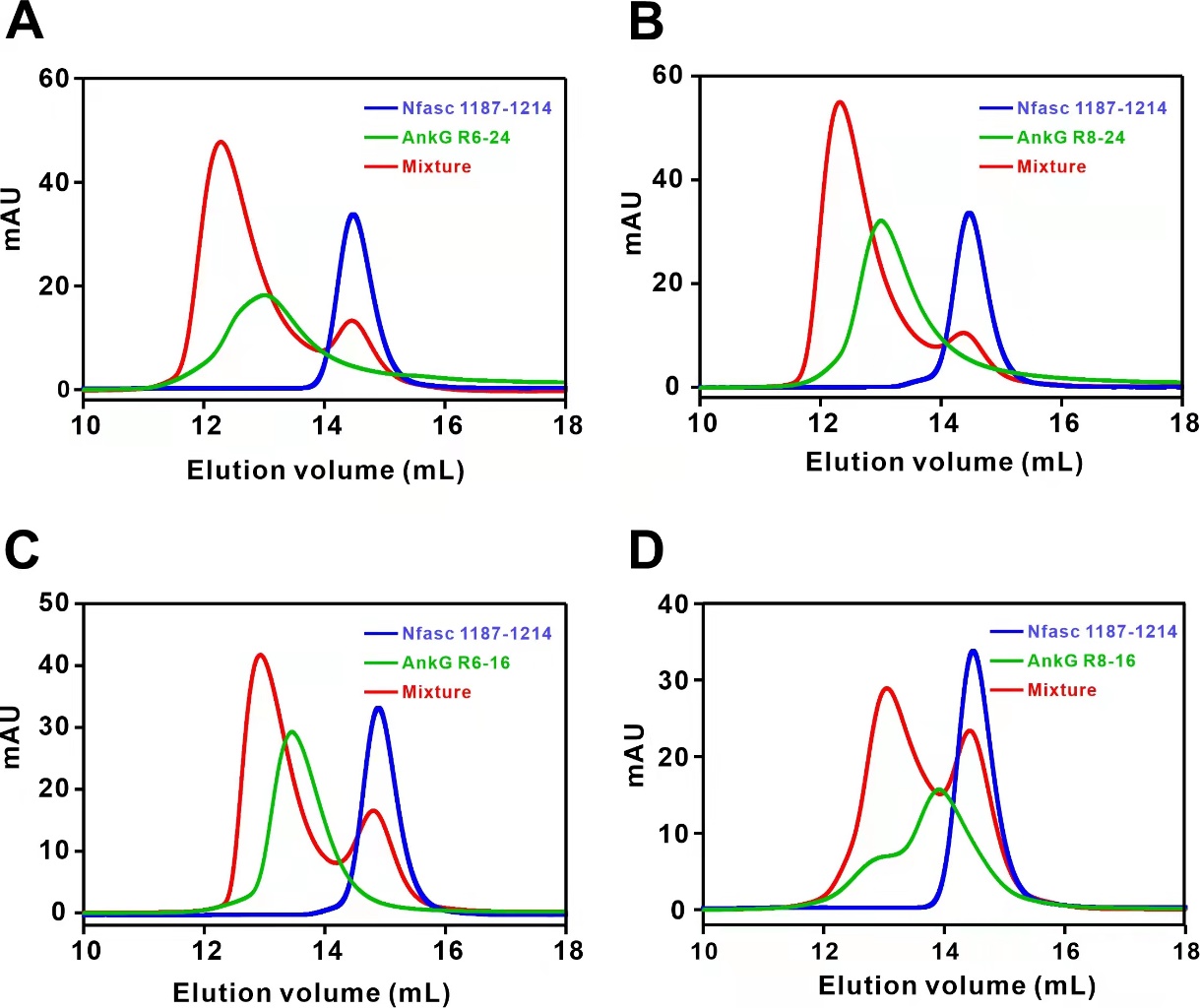
**

**Fig. S2** **AnkG ANK repeats binds to Nfasc ABD.** (A-D) Analytical gel filtration analysis showing that Nfasc ABD (residues 1187-1214) interacts with AnkG ANK repeats R6-24 (A), R8-24 (B), R6-16 (C), and R8-16 (D).

**
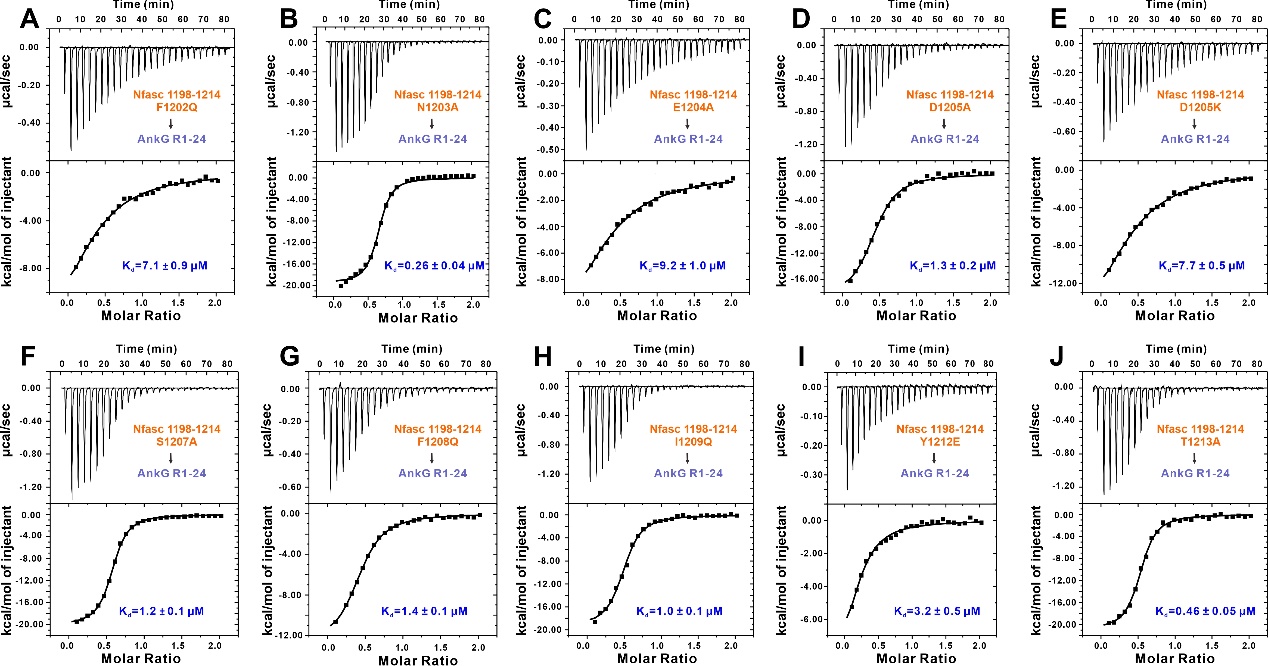
**

**Fig. S3** **Nfasc mutations decrease the binding affinities between Nfasc and AnkG.** ITC-based measurements of the binding affinities of various Nfasc 1198-1214 variants and AnkG R1-24.

**
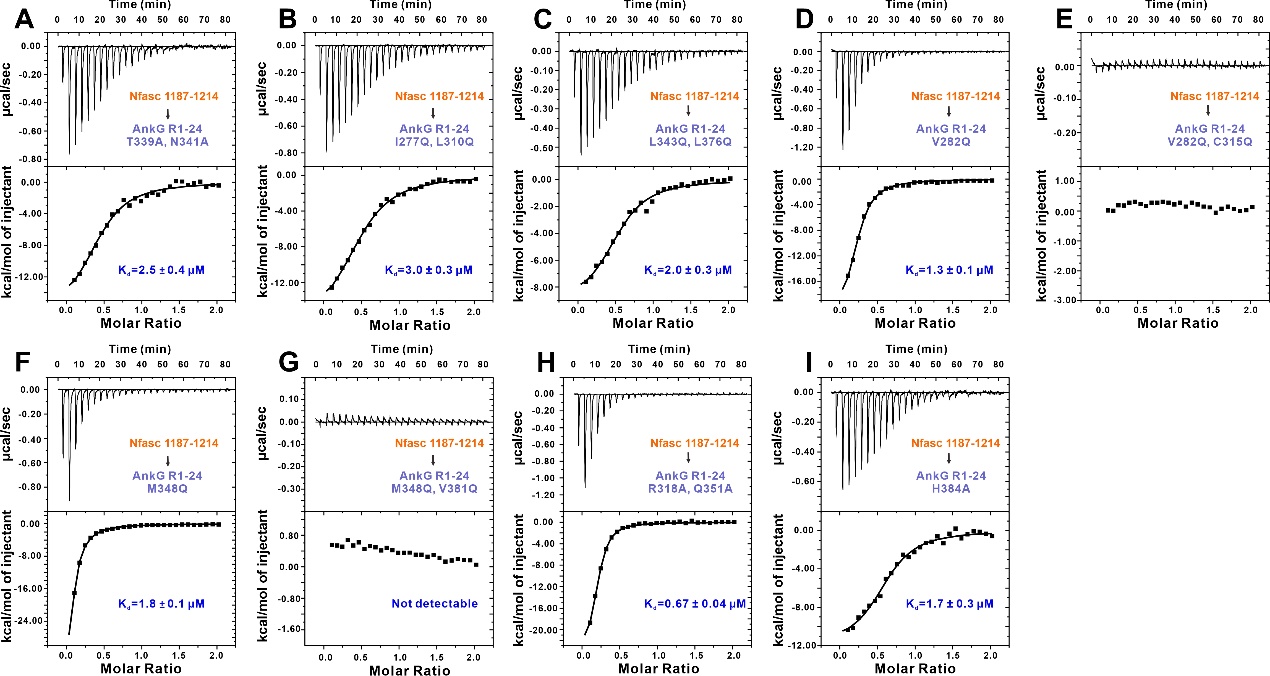
**

**Fig. S4** **AnkG ANK repeats mutations decrease the binding affinities between Nfasc and AnkG.** ITC-based measurements of the binding affinities of Nfasc 1187-1214 and various AnkG R1-24 variants.

**
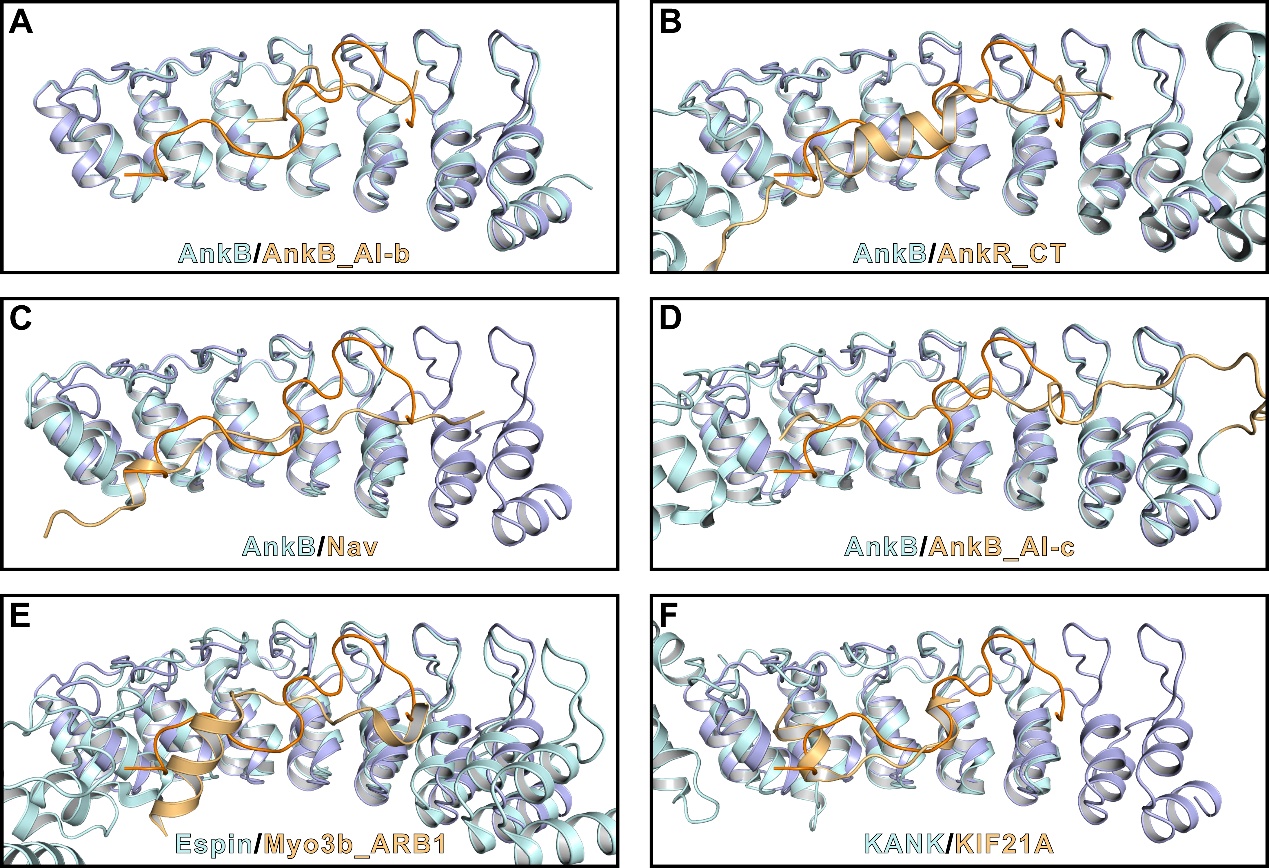
**

**Fig. S5** **Structure alignments of AnkG-Nfasc complex with other ANK repeats containing proteins in complex with respective cytoplasmic peptides.** (A) Structure alignment of AnkG-Nfasc complex and AnkB/AnkB AI-b complex, PDB ID: 5Y4E. (B) Structure alignment of AnkG-Nfasc complex and AnkB/AnkR_CT complex, PDB ID: 4RLV. (C) Structure alignment of AnkG-Nfasc complex and AnkB/Nav1.2 complex, PDB ID: 4RLY. (D) Structure alignment of AnkG-Nfasc complex and AnkB/AnkB AI-c complex, PDB ID: 5Y4F. (E) Structure alignment of AnkG-Nfasc complex and Espin/Myo3b_ARB1 complex, PDB ID: 5ET1. (F) Structure alignment of AnkG-Nfasc complex and KANK1/KIF21A complex, PDB ID: 5YAY. The color schemes are in line with the corresponding proteins in each individual panel.

**
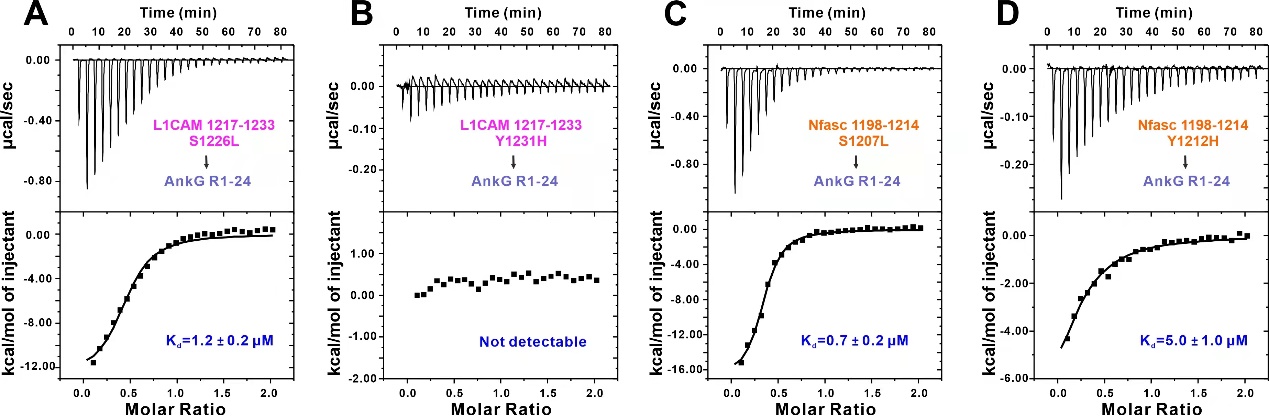
**

**Fig. S6** **L1 Syndrome-associated mutations decrease the binding affinities between L1CAM and AnkG.** (A, B) ITC-based measurements of the binding affinities between L1CAM 1217-1233 S1226L (A) or L1CAM 1217-1233 Y1231H (B) and AnkG R1-24. (C, D) ITC-based measurements of the binding affinities between Nfasc 1198-1214 S1207L (C) or Nfasc 1198-1214 Y1212H (D) and AnkG R1-24.


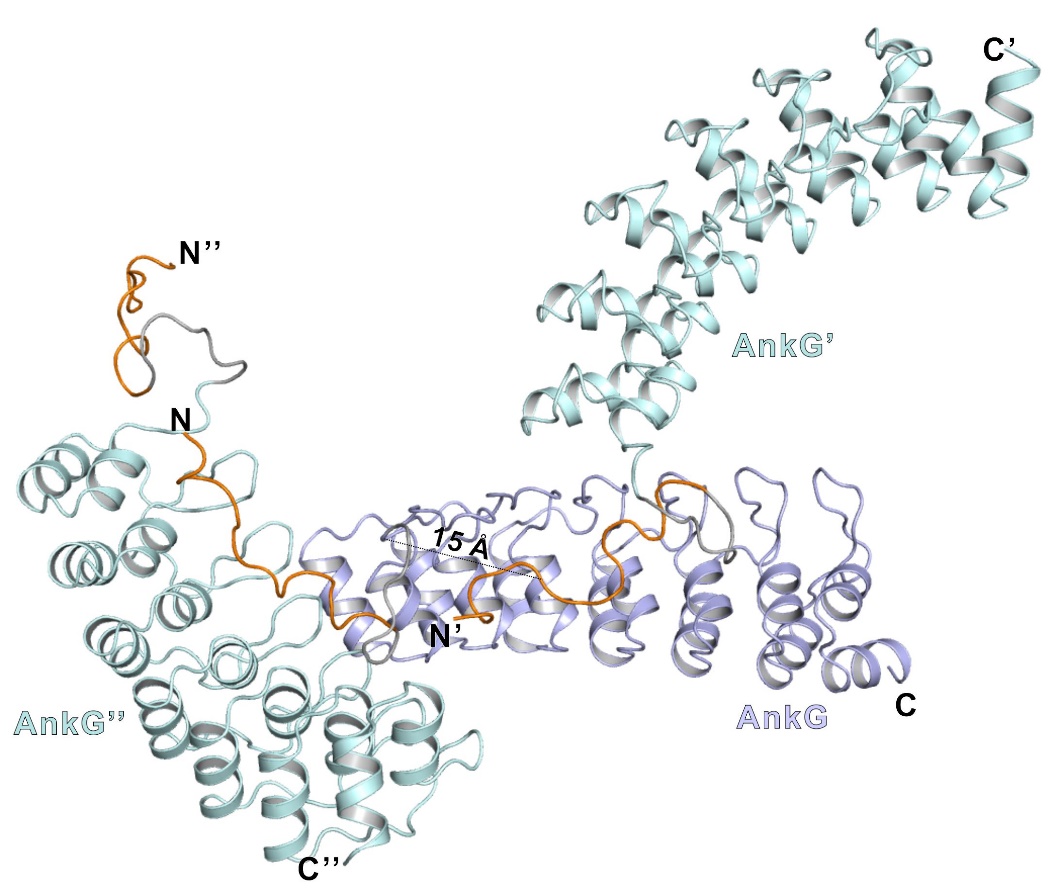


**Fig. S7** **Asymmetric units of the AnkG-Nfasc complex.** Ribbon representation model showing the asymmetric units of the AnkG-Nfasc complex. In this drawing, AnkG is shown in light blue and cyan, and Nfasc is shown in orange. Nfasc ABD bound to another AnkG molecule from the neighboring asymmetric unit, forming an intermolecular interaction.

**Supplementary figure legends**

**Fig. S1** **The binding region mapping between AnkG and Nfasc.** (A-E) ITC-based measurements of the binding affinities of Nfasc 1187-1214 and various AnkG ANK repeats including R6-24 (A), R8-24 (B), R1-5 (C), R1-7 (D), and R17-24 (E). (F, G) ITC-based measurements of the binding affinities between different Nfasc fragments and AnkG R1-24. The K_d_ error is the fitting error obtained using one site binding kinetics model in Origin 7.0 to fit the ITC data.

**Fig. S2** **AnkG ANK repeats binds to Nfasc ABD.** (A-D) Analytical gel filtration analysis showing that Nfasc ABD (residues 1187-1214) interacts with AnkG ANK repeats R6-24 (A), R8-24 (B), R6-16 (C), and R8-16 (D).

**Fig. S3** **Nfasc mutations decrease the binding affinities between Nfasc and AnkG.** ITC-based measurements of the binding affinities of various Nfasc 1198-1214 variants and AnkG R1-24.

**Fig. S4** **AnkG ANK repeats mutations decrease the binding affinities between Nfasc and AnkG.** ITC-based measurements of the binding affinities of Nfasc 1187-1214 and various AnkG R1-24 variants.

**Fig. S5** **Structure alignments of AnkG-Nfasc complex with other ANK repeats containing proteins in complex with respective cytoplasmic peptides.** (A) Structure alignment of AnkG-Nfasc complex and AnkB/AnkB AI-b complex, PDB ID: 5Y4E. (B) Structure alignment of AnkG-Nfasc complex and AnkB/AnkR_CT complex, PDB ID: 4RLV. (C) Structure alignment of AnkG-Nfasc complex and AnkB/Nav1.2 complex, PDB ID: 4RLY. (D) Structure alignment of AnkG-Nfasc complex and AnkB/AnkB AI-c complex, PDB ID: 5Y4F. (E) Structure alignment of AnkG-Nfasc complex and Espin/Myo3b_ARB1 complex, PDB ID: 5ET1. (F) Structure alignment of AnkG-Nfasc complex and KANK1/KIF21A complex, PDB ID: 5YAY. The color schemes are in line with the corresponding proteins in each individual panel.

**Fig. S6** **L1 Syndrome-associated mutations decrease the binding affinities between L1CAM and AnkG.** (A, B) ITC-based measurements of the binding affinities between L1CAM 1217-1233 S1226L (A) or L1CAM 1217-1233 Y1231H (B) and AnkG R1-24. (C, D) ITC-based measurements of the binding affinities between Nfasc 1198-1214 S1207L (C) or Nfasc 1198-1214 Y1212H (D) and AnkG R1-24.

**Fig. S7** **Asymmetric units of the AnkG-Nfasc complex.** Ribbon representation model showing the asymmetric units of the AnkG-Nfasc complex. In this drawing, AnkG is shown in light blue and cyan, and Nfasc is shown in orange. Nfasc ABD bound to another AnkG molecule from the neighboring asymmetric unit, forming an intermolecular interaction.
